# Dynamin-Like Protein B of *Dictyostelium* Contributes to Cytokinesis Cooperatively with Other Dynamins

**DOI:** 10.3390/cells8080781

**Published:** 2019-07-26

**Authors:** Koushiro Fujimoto, Masahito Tanaka, A.Y. K. Md. Masud Rana, Md. Golam Sarowar Jahan, Go Itoh, Masatsune Tsujioka, Taro Q. P. Uyeda, Shin-ya Miyagishima, Shigehiko Yumura

**Affiliations:** 1 Graduate School of Sciences and Technology for Innovation, Yamaguchi University, Yamaguchi 753-8511, Japan; 2 Institute of Food and Radiation Biology, AERE, Bangladesh Atomic Energy Commission, Dhaka 1000, Bangladesh; 3 Department of Biochemistry and Molecular Biology, Faculty of Science, University of Rajshahi, Rajshahi 6205, Bangladesh; 4 Department of Molecular Medicine and Biochemistry, Akita University Graduate School of Medicine, Akita 010-8543, Japan; 5 Department of Pathological Cell Biology, Medical Research Institute, Tokyo Medical and Dental University, Tokyo 113-8510, Japan; 6 Department of Physics, Faculty of Science and Technology, Waseda University, Tokyo 169-8555, Japan; 7 Department of Gene Function and Phenomics, Center for Frontier Research, National Institute of Genetics, Shizuoka 411-8540, Japan

**Keywords:** actin, contractile ring, cytokinesis, dynamin, endocytosis

## Abstract

Dynamin is a large GTPase responsible for diverse cellular processes, such as endocytosis, division of organelles, and cytokinesis. The social amoebozoan, *Dictyostelium discoideum*, has five dynamin-like proteins: dymA, dymB, dlpA, dlpB, and dlpC. DymA, dlpA, or dlpB-deficient cells exhibited defects in cytokinesis. DlpA and dlpB were found to colocalize at cleavage furrows from the early phase, and dymA localized at the intercellular bridge connecting the two daughter cells, indicating that these dynamins contribute to cytokinesis at distinct dividing stages. Total internal reflection fluorescence microscopy revealed that dlpA and dlpB colocalized at individual dots at the furrow cortex. However, dlpA and dlpB did not colocalize with clathrin, suggesting that they are not involved in clathrin-mediated endocytosis. The fact that dlpA did not localize at the furrow in dlpB null cells and vice versa, as well as other several lines of evidence, suggests that hetero-oligomerization of dlpA and dlpB is required for them to bind to the furrow. The hetero-oligomers directly or indirectly associate with actin filaments, stabilizing them in the contractile rings. Interestingly, dlpA, but not dlpB, accumulated at the phagocytic cups independently of dlpB. Our results suggest that the hetero-oligomers of dlpA and dlpB contribute to cytokinesis cooperatively with dymA.

## 1. Introduction

The dynamin superfamily is a large GTPase family that is responsible for diverse cellular processes, including various membrane-remodeling events, such as the fusion and fission of intracellular trafficking vesicles and fusion and fission of large organelles, including mitochondria, chloroplasts, and peroxisomes. Other dynamin superfamily proteins are involved in membrane independent viral resistance of host cells [1,2]. Recent studies have shown that dynamin also contributes to cytokinesis. Mammals have three classical dynamins and several dynamin-like proteins. In HeLa cells, dynamin 2 localizes at the spindle midzone and the subsequent intercellular bridge, suggesting an important role of this protein in the final separation of dividing cells [3,4]. In zebrafish blastomeres, dynamin 2 localizes at the cleavage furrow, contributing to cytokinesis through furrow-specific endocytosis [5]. In *Caenorhabditis elegans,* dynamin (Dyn-1) localizes at the cleavage furrow and accumulates at the midbody of dividing embryos. RNA interference silencing of *dyn-1* produced a marked defect in the late stage of cytokinesis [6]. The *Drosophila* dynamin homolog, *shibire*, localizes to sites of membrane invagination during cellularization, which is an alternate form of cytokinesis [7]. The dynamin superfamily also contributes to cytokinesis in plants. The soybean DRP1 homolog, phragmoplastin, was the first dynamin shown to be involved in the cytokinesis [8]. *Arabidopsis* has 16 dynamin-like proteins, which are grouped into six subfamilies, of which DRP1, DRP2, and DRP5 are involved in cytokinesis [9,10,11]. Recently, a dynamin-like protein has been reported to contribute to cytokinesis in green algae [12]. In addition, in *Streptomyces*, dynamin-like proteins (DynA and DynB) stabilize FtsZ rings, thus playing an important role in cytokinesis [13]. However, the molecular mechanism by which these dynamins contribute to cytokinesis is still elusive.

*Dictyostelium* is a model organism to examine the mechanism of cell migration, chemotaxis, and cytokinesis. *Dictyostelium discoideum* has five dynamin-like proteins: dymA, dymB, dlpA, dlpB, and dlpC [11,14,15]. DymA and dymB have three domains—a GTPase domain, a middle domain, and GTPase effector domain (GED) [14,16]. An additional QNS (glutamine, serine, and asparagine) domain is present in dymA, and a QPS (glutamine, proline, and serine) domain is present in dymB [14,16]. DlpA, dlpB, and dlpC have a GTPase domain near the N-terminal but do not contain other specific domains. Phylogenetic analysis places dymA and dymB in the same branch as the yeast proteins, Vps1p and Dnm1p, and the mammalian protein DRP1. The members of this group appear to play a role in peroxisomal and mitochondrial division, vesicle trafficking, and cytokinesis [11,14,16]. DlpA, dlpB, and dlpC are grouped with the plant dynamin-related proteins DRP5A and DRP5B, which are involved in cytokinesis and chloroplast division [11]. In previous reports, *Dictyostelium* mutant cells lacking dymA showed alterations in mitochondrial, nuclear, and endosomal morphology, as well as a defect in fluid-phase uptake [16]. However, more recently, Schimmel et al. have reported that dymA and dymB are not essential for mitochondrial fission or fusion [15]. DymB depletion affects many aspects of cell motility, cell–cell and cell–substratum adhesion, resistance to osmotic shock, and fatty acid metabolism [14]. In addition, we have shown that dlpA and dymA localize at the furrow of dividing cells [11,17].

*Dictyostelium* cells have four modes of cytokinesis—cytokinesis A, B, C, and D [18,19,20,21]. Cytokinesis A depends on the contractile ring, cytokinesis B depends on the traction force of both the daughter cells, cytokinesis C is independent of cell cycle, and cytokinesis D is mediated by midwifery of other cells. Myosin II null cells divide by the traction force (cytokinesis B) without the constriction power of myosin II [22]. However, wild-type cells use both the constriction of contractile ring (cytokinesis A) and traction force (cytokinesis B) on the adherent culture condition [19]. The molecular mechanism underlying the regulation of actin and myosin II in the formation and maintenance of the contractile ring is still unsolved [23].

Here, we show the role of dlpB in *Dictyostelium* cytokinesis. DlpA and dlpB colocalized at the furrow from the initial furrowing and dymA accumulated at the same site in the last stage of cytokinesis, suggesting that these dynamins play distinct roles in cytokinesis. Furthermore, we found that hetero-oligomerization of dlpA and dlpB is required for them to associate with the furrow. These hetero-oligomers are involved in the stabilization of actin filaments in the furrow, but not in clathrin-mediated endocytosis. Interestingly, we found that dlpA also accumulates at the phagocytic cups independently of dlpB. We suggest that the hetero-oligomers of dlpA and dlpB contribute to cytokinesis cooperatively with dymA.

## 2. Materials and Methods

### 2.1. Cell Culture

*Dictyostelium discoideum* wild-type (AX2) cells and all mutant cells were cultured in HL5 medium (1.3% bacteriological peptone, 0.75% yeast extract, 85.5 mM D-glucose, 3.5 mM Na_2_HPO_4_, and 3.5 mM KH_2_PO_4_, pH 6.4) at 22 °C. Cells were cultured in suspension at 150 rpm or in plastic dishes. To synchronize the cell cycle and increase the number of mitotic cells, cells were cultured at 10 °C for 16 h and treated with 10 µM thiabendazole at 22 °C for 3.5 h. *Escherichia coli* (B/r) was cultured in HL5 medium in suspension and washed with 15 mM Na–K phosphate buffer (pH 6.3) by centrifugation.

### 2.2. Plasmid Construction and Transformation

Expression vectors containing GFP-lifeact, GFP-dlpA, GFP-dlpB, GFP-dymA, mCherry-dlpB, and GFP-clathrin (light chain) were transformed into wild-type and dynamin mutant cells by electroporation or laserporation as described previously [24,25]. Positive cells were selected using 10 µg/mL G418 (Wako, Osaka, Japan) for GFP-lifeact, GFP-dlpA, GFP-dlpB, GFP-dymA, and GFP-clathrin, and 10 µg/mL blasticidin (Wako) for mCherry-dlpB. Full length GFP-dlpB, GFP-dlpB, GFP-fragments, and GFP-dymA constructs were generated by cloning BamHI digested, PCR-amplified products into the pA15GFP vector. The mCherry-dlpB construct was generated by cloning BamH1 and XhoI digested, PCR-amplified product into the mCherry/pDdBr vector. GFP-clathrin construct was generated by cloning BamHI and SacI digested, PCR-amplified product into the GFP/pDNeo vector. Knockout mutants, dlpA^-^, dlpB^-^, and dymA^-^, and the GFP-dlpA constructs have been previously described [11,17]. To generate double knockout mutant dlpA^-^/dlpB^-^, the Cre-*lox*P system was used [26]. Briefly, *lox*P, including blasticidin-resistance gene (BSR), was inserted into the pBCSK(+) vector containing PCR-amplified dlpA sequence, and the knockout cells were selected by blasticidin after electroporation. To remove the BSR, these dlpA^-^ cells were transformed with pACT-Cre, a Cre expressing vector (provided by Dr. H. Kuwayama), and then selected by G418. In the same way, the *dlpB* gene was also knocked out in dlpA^-^ cells. Myosin II null cells (HS1) were originally generated by Manstein et al. [27].

### 2.3. Antibodies

Polyclonal antibodies against dlpA and dymA were described previously [17]. Polyclonal antibodies against dlpB were newly generated. A synthetic polypeptide (aa 790–808) of dlpB (CNYKKYSQSFSHPFPSAVRN) was used for immunizing rabbits, by a custom service (Sigma-Aldrich, Tokyo, Japan). These antibodies were absorbed with fixed dlpB^-^ cells, as previously described [28], and used after 100-fold dilution for immunostaining and 1000-fold dilution for western blot.

### 2.4. Cells Preparations for Microscopy

Nuclei were stained with 4,6-diamidino-2-phenylindole (DAPI) (Sigma-Aldrich, Tokyo, Japan) after fixation with 2.5% formaldehyde in 15 mM Na/K phosphate buffer as previously described [29].

For immunostaining with anti-dlpB antibodies, cells were fixed by agar-overlay method as described previously [30]. The fixed cells were then incubated with anti-dlpB antibodies and later with Alexa 488-conjugated secondary antibodies (Thermo Fisher Scientific, Tokyo, Japan).

To stain Triton X-100-extracted cells, cells were overlaid with an agarose sheet and permeabilized by adding 10 µL of 2× Triton buffer (10 mM PIPES, 100 mM NaCl, 10 mM EGTA, 10 mM EDTA, 4 mM NaF, 0.2 mM phenylmethylsulfonyl fluoride, 2 mM dithiothreitol (DTT), 10 mM benzamidine, and 1% Triton X-100, pH 7.5) for 5 min and then washed in wash buffer (5 mM PIPES, 15 mM NaCl, 2 mM MgCl_2_, 0.2 mM DTT, and 0.1% NaN_3_, pH 7.5). The cells were subsequently stained with 50 ng/mL fluorescein isothiocyanate (FITC)-conjugated phalloidin (Sigma-Aldrich) and 0.1 mg/mL TRITC-DNase I (Molecular Probes, Eugene, OR, USA) for 30 min and then washed with the wash buffer.

Latrunculin A (1 µM final concentration, Sigma-Aldrich) was applied on the agar surface of the agar-overlaid cells.

For phagocytosis experiments, bacteria were mixed with cells expressing GFP-dynamins and then mildly pressed with an agar block. Time-lapse images were acquired at an interval of 5 or 10 s.

### 2.5. Microscopy

The DAPI-stained cells were observed using a fluorescence microscope (TE 300, Nikon, Japan) equipped with a regular UV filter set. Fluorescence images of live cells expressing GFP-dynamins and GFP-clathrin were acquired by a confocal microscope (LSM510, Zeiss, Germany) or a custom-made total internal reflection fluorescence (TIRF) microscope [31].

Traction force exerted by dividing cells was measured as previously described [22]. Briefly, cells were placed on an elastic silicone substratum with fluorescent red beads and observed using a DeltaVision microscope system (GE Healthcare, Little Chalfont, UK). To acquire the initial position-image of the beads, 10% sodium azide was added to kill the cells after the observation.

Interference reflection microscopy (IRM) was also simultaneously conducted using a DeltaVision microscope as previously described [22]. The cell-substratum adhesion area was measured using ImageJ software (http://rsbweb.nih.gov/ij/).

### 2.6. Fluorescence Recovery after Photobleaching (FRAP) Analysis

For FRAP experiments, wild-type and mutant cells expressing GFP-lifeact were agar-overlaid. Live imaging and photobleaching experiments were performed using the confocal microscope. The half-time of fluorescence recovery was calculated as described previously [32].

### 2.7. Immunoblotting and Co-Immunoprecipitation

For immunoblotting of the whole cell lysates, cells were directly lysed in 2× SDS sample buffer (0.125 M Tris-HCl, 4% sodium dodecyl sulfate, 20% glycerol, 0.2 M DTT, 0.02% bromophenol blue, pH 6.8). Alternatively, the cells were extracted with a buffer containing Triton X-100 and subjected to western blot, as described previously [17]. Briefly, the cells were extracted with Triton buffer (5 mM PIPES, 50 mM NaCl, 5 mM EGTA, 5 mM EDTA, 2 mM NaF, 0.1 mM phenylmethylsulfonyl fluoride, 1 mM DTT, 10 mM benzamidine, and 1% Triton X-100, pH 7.5) on ice for 15 min; the insoluble and soluble fractions were separated by centrifugation, and were subjected to western blot. The quantitative analysis was performed using the ImageJ software.

For co-immunoprecipitation experiments, magnetic beads (Dynabeads protein G, ThermoFisher) were conjugated with anti-dlpB antibodies in PBS (137 mM NaCl, 8 mM Na_2_HPO_4_, 1.47 mM KH_2_PO_4_, 2.7 mM KCl, pH 7.4) for 1 h. The synchronized mitotic cells or non-synchronized cells were lysed for 10 min in ice-cold lysis buffer (150 mM NaCl, 5 mM EGTA, 5 mM EDTA, 2.5 mM sodium pyrophosphate, 1 mM DTT, 1% protein inhibitor cocktail (Sigma-Aldrich), 1% Triton X-100, 20 mM Tris-HCl, pH 7.5). After centrifugation (15,000 rpm for 10 min), the supernatant was incubated with the antibodies-bound Dynabeads for 1 h. After washing five times with a wash buffer (the lysis buffer without Triton X-100), the beads were mixed with 2× SDS sample buffer, subjected to a standard SDS-PAGE, and then detected by western blot analysis using anti-dlpA and anti-dlpB antibodies.

### 2.8. Statistical Analysis

Statistical analysis was performed using GraphPad Prism 7 (GraphPad Software Inc., San Diego, CA, USA). Data were analyzed using Student’s *t*-test for comparison between two groups, or one-way ANOVA with Tukey’s multiple comparison test, and are presented as the mean ± standard deviation (SD).

## 3. Results

### 3.1. DlpA, DlpB, and DymA Contribute to Cytokinesis in Different Manners

**Figure 1:**
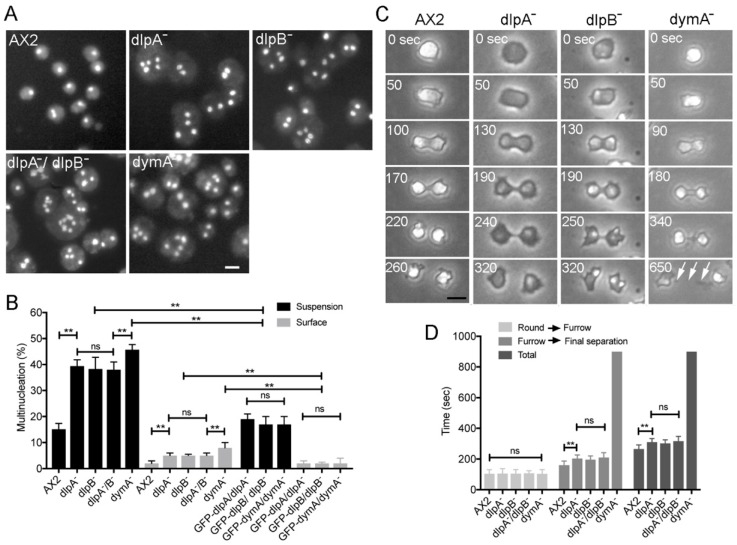
Three kinds of dynamin contribute to cytokinesis. (**A**) Typical fluorescence images of the nuclei of wild-type (AX2) cells and dynamin null mutant cell lines (dlpA^-^, dlpB^-^, dlpA^-^/dlpB^-^, and dymA^-^). Cells were stained with 4,6-diamidino-2-phenylindole (DAPI) after 3 days of culture in suspension. (**B**) Multinucleation of five cell lines and dynamin null cells transformed with the GFP-dynamins cultured on surface and in suspension (>500 cells, three independent experiments for each condition). (**C**) Typical phase contrast images of each cell line during cytokinesis on coverslips. DymA^-^ cells showed much longer intercellular bridges (arrows) and took a much longer time to separate. (**D**) The required time from the round stage to the initiation of furrowing, the time from the initiation of furrowing to the final separation, and the total time for the cell division for each cell line (>100 cells, three independent experiments for each). The time for dymA^-^ cells was considered as 20 min if it exceeded 20 min. Data are presented as mean ± SD and analyzed by one-way ANOVA with Tukey’s multiple comparison test. ** *p* ≤ 0.001; ns, not significant, *p* > 0.05. Bars, 10 µm.

*Dictyostelium discoideum* has five genes coding for dynamin-like proteins: *dymA*, *dymB*, *dlpA, dlpB*, and *dlpC*. For simplicity, here we will refer to dynamin-like proteins as dynamins. Previously, we and other groups have suggested that among these five dynamins, dlpA, dlpB, and dymA may contribute to cytokinesis [16,17]. We observed the nuclei in each knockout mutant (dlpA^-^, dlpB^-^, and dymA^-^) after the culture in suspension condition (Figure 1A). The three mutant cells became much larger than wild-type (A×2) cells and contained multiple nuclei, suggesting that these mutant cells have a defect in cytokinesis. A double knockout mutant in both dlpA and dlpB (dlpA^-^/dlpB^-^) also showed multinucleation. We tried to generate other double and triple mutants; however, the efforts were unsuccessful, suggesting that they could be lethal.

Figure 1B shows a summary of multinucleation of each mutant when cultured in suspension and on a surface. In both conditions, these mutants showed more multinucleation as compared to the wild-type cells; the multinucleation in the suspension culture was much more severe than that observed in adherent culture conditions. Interestingly, the double mutant dlpA^-^/dlpB^-^ cells showed a similar level of multinucleation as that of single mutants, suggesting that dlpA and dlpB might cooperatively contribute to the cytokinesis.

We observed each cell line during cell division on coverslips under phase contrast microscopy (Figure 1C). Typically, when *Dictyostelium* cells enter the mitotic phase, they stop migration, become round, elongate, and constrict the cleavage furrow to separate into two daughter cells. All mutant cells showed a similar morphological process as the wild-type cells; however, dlpA^-^ and dlpB^-^ cells appeared darker under the phase contrast microscopy. Remarkably, all mutants took a longer time to complete the final separation.

To determine the stage of cytokinesis at which the cells were delayed, the time required from the round stage to the initiation of furrowing, that from the initiation of furrowing to the final separation, and the total time for the cell division were examined in each mutant (Figure 1D). The time required from round stage to the initiation of furrowing of the mutants was not significantly different from that of wild-type cells. However, dlpA^-^, dlpB^-^, and dlpA^-^/dlpB^-^ cells took a significantly longer time for constricting the furrow. On the other hand, dymA^-^ cells exhibited a much longer intercellular bridge and took a longer time to separate (arrows in Figure 1C) (sometimes longer than 20 min).

### 3.2. Three Dynamins Localize at the Cleavage Furrow

Next, we examined the localization of the three dynamins in dividing cells. We have previously shown that dlpA and dymA localize at the furrow region [17]. Here, we observed the localization of dlpB and compared it with that of other dynamins in live cells. Each GFP-tagged dynamin was expressed in individual knockout mutant cells. Figure 1B shows the frequencies of multinucleation in these cells, indicating that the expression of the GFP-tagged protein rescued the defects in cytokinesis in both suspension and adherent culture conditions.

**Figure 2:**
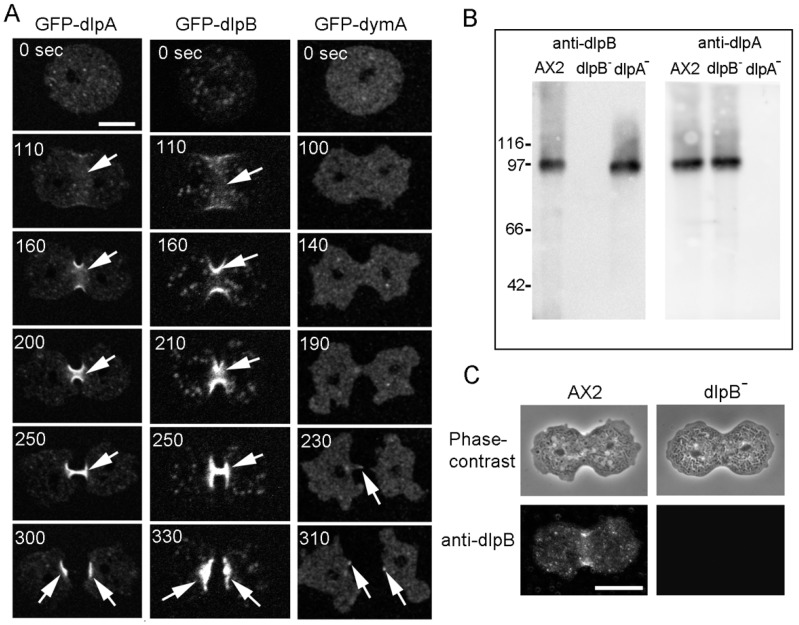
Three dynamins localize at the cleavage furrow. (**A**) Typical time courses of live images of GFP-dlpA, GFP-dlpB, and GFP-dymA during cell division. GFP was tagged to the C-terminus of each dynamin. Both GFP-dlpA and GFP-dlpB localized at the furrow from the initiation of furrowing to the final separation (arrows). On the other hand, a small amount of GFP-dymA localized at the connecting thread during the final separation. These observations were confirmed in at least 50 dividing cells for each cell line. (**B**) Western blots of whole-cell lysate of each cell line were probed with anti-dlpB and anti-dlpA antibodies. (**C**) Typical phase-contrast and immuno-fluorescence images of A×2 and dlpB^-^ cells stained with anti-dlpB antibody. Bars, 10 µm.

Figure 2A shows typical time course of fluorescence images of GFP-dlpA, GFP-dlpB, and GFP-dymA in the respective knock-out cell line during the cell division. Both GFP-dlpA and GFP-dlpB localized at the cleavage furrow from the initial stage of furrowing to the final separation. However, a small amount of GFP-dymA localized at the intercellular bridge only during the final separation.

We generated antibodies against dlpB and confirmed their specificity by western blot (Figure 2B). There was no detectable band corresponding to dlpB in dlpB^-^ cells. The anti-dlpA antibodies, which have been previously described [17], found a similar expression level of dlpA in dlpB^-^ cells to that of wild-type (A×2) cells. Similarly, dlpB was found to be expressed in a similar level in dlpA^-^ cells to that of wild type cells, suggesting that dlpA and dlpB were stable in each mutant cell.

Next, we immuno-stained wild-type and dlpB^-^ cells with the anti-dlpB antibody (Figure 2C). The fluorescence images showed that dlpB localized at the cleavage furrow of wild-type cells, although there was no detectable staining in dlpB^-^ cells, suggesting that the endogenous dlpB also localize at the furrow in wild-type cells. We have already confirmed that the endogenous dlpA and dymA show the same localization as the GFP-tagged proteins in the live cells [17].

Collectively, dlpA and dlpB might contribute to the furrowing and dymA might contribute to the final separation.

### 3.3. DlpA and DlpB Colocalize at the Cleavage Furrow

**Figure 3:**
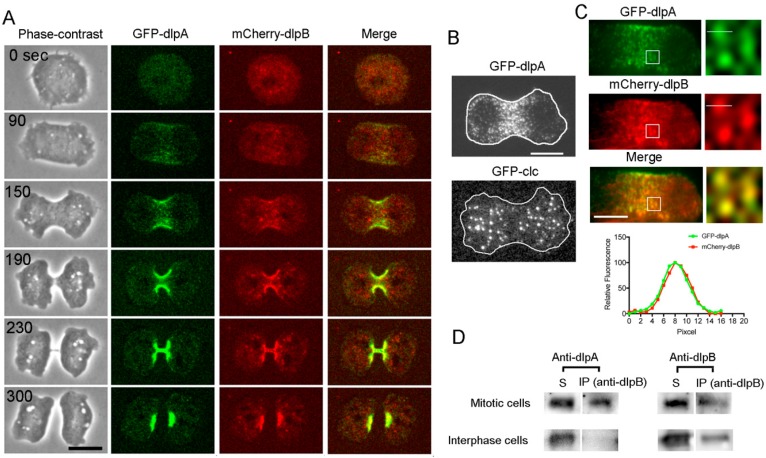
DlpA and dlpB colocalize at the cleavage furrow. (**A**) A typical time course of fluorescence images of a dividing cell simultaneously expressing GFP-dlpA (green) and mCherry-dlpB (red). The merged images (Merge) show that both proteins accumulated at the same place at the same timing. These observations were confirmed in 18 dividing cells. (**B**) Typical total internal reflection fluorescence (TIRF) images of GFP-dlpA and GFP-clathrin light chain (GFP-clc). The outlines of the cells are indicated in white. (**C**) Typical TIRF images of GFP-dlpA and mCherry-dlpB in the same dividing cell. The right panels show enlarged images of the boxes in the left panels. The graph shows the fluorescence intensity profile on the white line across the single dot. (**D**) Co-immunoprecipitation assay. After anti-dlpB antibodies-bound magnetic beads were mixed with the lysate of partially synchronized wild-type cells, and the immunoprecipitated proteins (IP) and the supernatant proteins (S) were detected by western blot using anti-dlpA and anti-dlpB antibodies, respectively. The amount of dlpA in the IP fraction of synchronized (mitotic) cells was 58 ± 8% of that in supernatant (*n* = 3), but only slight amount of dlpA was detected in non-synchronized (interphase) cell lysate. Bars, 10 µm.

GFP-dlpA and mCherry-dlpB were simultaneously observed in dlpA^-^/dlpB^-^ cells. Figure 3A shows a typical time course of fluorescence images. The merged images (Merge) indicate that both proteins simultaneously accumulate at the same place.

Next, the cells expressing GFP-dlpA were observed using total internal reflection fluorescence (TIRF) microscopy, which enables the selective visualization of about 100 nm (depth) above the coverslip, covering the thickness of the cell cortex. Figure 3B shows a typical TIRF image of GFP-dlpA in a dividing cell. Many individual dots consisting of dlpA were observed at the furrow cortex.

The scission of newly formed vesicles from the membrane, such as in endocytosis, by the dynamin is well established. To examine the possibility that dlpA is also involved in endocytosis, we compared the localization of dlpA with that of clathrin, which plays a major role in the formation of coated vesicles. Although GFP-clathrin also appeared as dots in the cell membrane when visualized by the TIRF microscope, it did not accumulate at the furrow (Figure 3B). Thus, it is unlikely that dlpA is involved in the clathrin-mediated endocytosis.

Next, GFP-dlpA and mCherry-dlpB were simultaneously observed using TIRF microscopy (Figure 3C). The fluorescence intensity profile across the single dot (white line in TIRF images) indicates that both proteins were found to colocalize at the same dots. As dynamin is generally known to form oligomers [33], dlpA and dlpB may form a hetero-oligomer as a functional unit.

To investigate this possibility, co-immunoprecipitation assay was performed. The anti-dlpB antibody-bound magnetic beads were mixed with the cell lysate of the partially-synchronized mitotic cells and the co-sedimented proteins were subjected to western blot analysis using anti-dlpA and anti-dlpB antibodies (Figure 3D). A substantial amount of dlpA was detected in the sedimented fraction. However, only a slight amount of dlpA was detected when using non-synchronized (interphase) cell lysate (if the total cell division time is 5 min and the total doubling time is 8 hr, mitotic cells account for 1.0% in the non-synchronized cells). These results indicate that dlpA and dlpB directly or indirectly bind to each other, depending on the cell cycle.

### 3.4. Both DlpA and DlpB are Required for their Localization to the Furrow

**Figure 4:**
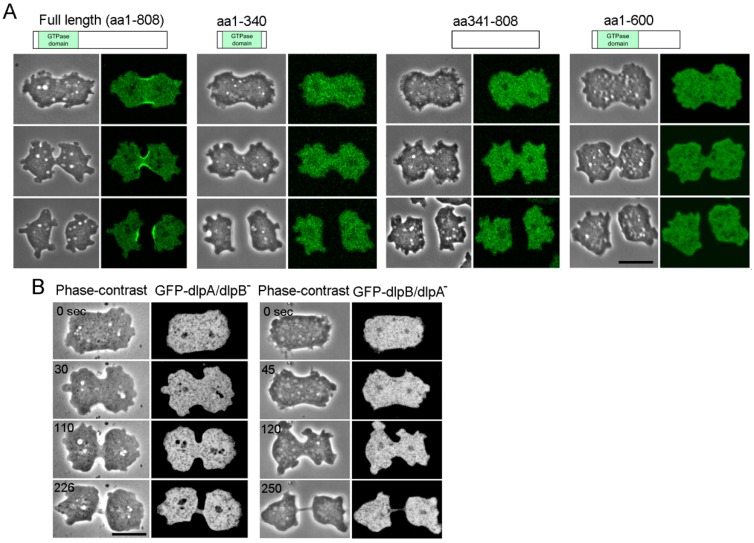
Both dlpA and dlpB are required for their localization to the furrow. (**A**) Domain analysis of dlpB for its localization at the furrow. GFP-fusion proteins with the GTPase domain (aa 1–340), a fragment without the GTPase domain (aa 341–808), and a fragment without the C-terminal domain (aa 1–600) were expressed in dlpB^-^ cells. Typical phase contrast and fluorescence images during cytokinesis. Only full length dlpB localized at the furrow. (**B**) When GFP-full length dlpB was expressed in dlpA^-^ cells, dlpB did not localize at the furrow (right panel). When GFP-full length dlpA was expressed in dlpB^-^ cells, dlpA did not localize at the furrow (left panel). These observations were confirmed in more than 25 cells for each case. Bars, 10 µm.

To examine which part of the dlpB molecule is required for its localization to the furrow, domain analysis was performed. DlpB contains a GTPase domain, which is a common characteristic domain of the dynamin family. A GFP-GTPase domain (aa 1–340), a GFP-fragment without the GTPase domain (aa 341–808), and a GFP-fragment without the C terminal domain (aa 1–600) were expressed in dlpB^-^ cells. None of these fragments showed any localization at the furrow (Figure 4A). Therefore, full length of dlpB is indispensable for its localization at the furrow.

Next, GFP-full length dlpB was expressed in dlpA^-^ cells. Interestingly, dlpB did not localize at the furrow. Conversely, GFP-dlpA did not localize at the furrow in dlpB^-^ cells (Figure 4B). Therefore, both, dlpA and dlpB are required for their localization at the furrow.

Given these observations, we concluded that dlpA and dlpB accumulate to the furrow as a complex, presumably in a form of hetero-oligomer.

### 3.5. DlpA and DlpB Associate with Actin Filaments at the Cleavage Furrow

**Figure 5:**
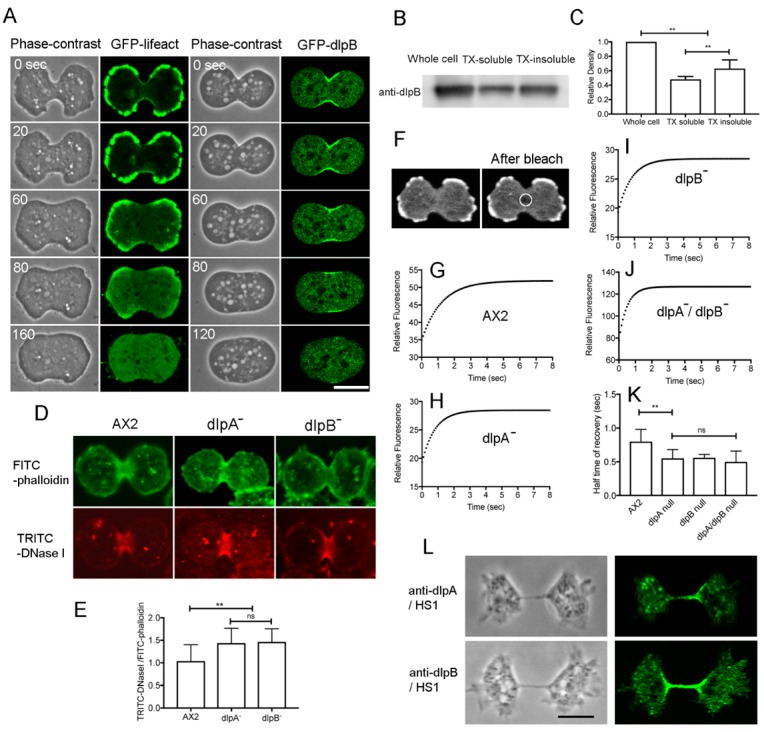
DlpA and dlpB stabilize actin filaments at the cleavage furrow. (**A**) Typical time courses of A×2 cell expressing GFP-lifeact and dlpB^-^ cell expressing GFP-dlpB upon latrunculin A application. (**B**) Western blot of the whole cell, Triton-insoluble cytoskeleton (T×-insoluble), and soluble fraction (T×-soluble) using an anti-dlpB antibody. (**C**) The relative amount of dlpB in the whole cell, T×-insoluble fraction, and T×-soluble fraction (three independent experiments). (**D**) Typical fluorescence images of the Triton-extracted wild-type dlpA^-^ and dlpB^-^ cells that were simultaneously stained with fluorescein isothiocyanate (FITC)-phalloidin and TRITC-DNase I. (**E**) Quantitative comparison of the relative fluorescence of DNase I among the three cell lines. The fluorescence intensity of TRITC-DNase I was divided by that of FITC-phalloidin after the subtraction of the background (>20 cells, three independent experiments). (**F**) Photobleaching experiments were carried out at the furrow (circle) of wild-type expressing GFP-lifeact. The two panels show fluorescence images taken before and after photobleaching at the furrow. To observe only the cortex, the optical section was set at 1.0 µm. (**G–J**) Representative curves of fluorescence recovery of GFP-lifeact at the furrow in wild-type (**G**), dlpA^-^ (**H**), dlpB^-^ (**I**), and dlpA^-^/dlpB^-^ cells (**J**). (**K**) Comparisons of half time of recovery among the cell lines (>20 cells, three independent experiments). (**L**) Typical fluorescence images of myosin II null cells (HS1) after staining with anti-dlpA and anti-dlpB antibodies. Data in panels C, E, and K are presented as mean ± SD and analyzed by one-way ANOVA with Tukey’s multiple comparison test. ** *p* ≤ 0.001; ns, not significant, *p* > 0.05. Bars, 10 µm.

Previously, we have suggested that dlpA may associate with actin filaments as the lack of dlpA caused severe fragmentation of actin filaments in the contractile ring [17]. Hence, we examined whether dlpB associates with actin filaments in the contractile ring as well. When latrunculin A, a depolymerizer of actin filaments, was added to dividing cells expressing GFP-lifeact, the actin filaments disappeared, which caused loosing of the furrow and failure of cytokinesis (Figure 5A, left). GFP-dlpB also delocalized from the cell membrane after latrunculin A treatment, suggesting that dlpB associates with actin filaments (Figure 5A, right). Our previous observations showed that GFP-dlpA was also delocalized from the cleavage furrow upon latrunculin A treatment [17].

To confirm the association between dlpB and actin filaments, the synchronized cells were extracted with a buffer containing Triton X-100. The insoluble actin cytoskeletons were subjected to western blot analysis using an anti-dlpB antibody. After the extraction, a substantial amount of dlpB was still present in the cytoskeleton (63.0 ± 12%, Figure 5B,C). DlpA also remained in the actin cytoskeleton, as described previously [17].

Collectively, both, dlpA and dlpB directly or indirectly associate with actin filaments at the cleavage furrow.

### 3.6. DlpA and dlpB Stabilize the Actin Filaments at the Cleavage Furrow

To examine the fragmentation of the actin filaments in the contractile rings of mutant cells, we carried out TRITC-DNase I staining. DNase I binds to the pointed ends of actin filaments as well as the subdomains II and IV of monomeric actin [34,35]. If actin filaments are fragmented at the furrow, there would be more free ends of actin filaments available for binding to TRITC-DNase I. After lysing the cells with Triton X-100, the insoluble cytoskeleton was simultaneously stained with TRITC-DNase I and FITC-phalloidin. Monomeric actin is substantially extracted under these conditions. Interestingly, TRITC-DNase I mainly stained the furrow regions, whereas FITC-phalloidin stained the cell cortex as well as the furrow (Figure 5D). The fluorescence intensity of TRITC-DNase I relative to that of FITC-phalloidin at the furrow was significantly higher (*p* ≤ 0.001) in dlpA and dlpB mutant cells than in wild-type cells (Figure 5E), which suggests that actin filaments are fragmented at the furrow region of the mutant cells. Therefore, these dynamins may contribute to the stabilization of actin filaments in the contractile rings.

To examine this possibility, fluorescence recovery after photobleaching (FRAP) was carried out at the furrow of each mutant cell expressing GFP-lifeact (Figure 5F). Previously, we have used GFP-lifeact for the estimation of the turnover of actin filaments and proved that the turnover of GFP-lifeact reflects the turnover of actin filaments [36]. However, a recent report has described that GFP-lifeact can modify the turnover of actin filaments depending on the expression levels [37]. We compared the expression level of each mutant from western blot using anti-GFP antibodies and found that there were no significant differences (A×2:dlpA^-^:dlpB^-^ = 1.00:1.09 ± 0.10:0.98 ± 0.11, *p* > 0.05, three independent experiments). Therefore, we considered ourselves to be able to compare the half-time of recovery among mutants. The half-time of fluorescence recovery was significantly shorter in the mutant cells than in the wild-type cells (Figure 5G–K).

Taken together, these results suggest that dlpA and dlpB stabilize the actin filaments by suppressing the extent of filament fragmentation in the contractile rings. The actin turnover rates were almost the same in the double mutant as in the single mutants, again suggesting that dlpA and dlpB function cooperatively.

Myosin II also accumulates to the cleavage furrow and contributes to the constriction of the furrow. Myosin II can cut actin filaments and enhance their turnover in the contractile rings [31]. Thus, we examined whether dlpA and dlpB localize at the cleavage furrow in myosin II null cells (HS1). Figure 5L shows typical immunofluorescence images of HS1 cells stained with anti-dlpA and anti-dlpB antibodies, indicating that dlpA and dlpB localize at the cleavage furrow independently of myosin II. In addition, this observation suggests that these dynamins contribute to cytokinesis B (traction-mediated cytokinesis) as well as cytokinesis A (contractile ring-dependent cytokinesis).

### 3.7. Dynamins Contribute to Cell-Substratum Adhesion and Traction Force

Under the phase-contrast microscope, dividing dlpA^-^ and dlpB^-^ cells appeared significantly darker than the wild-type cells (Figure 1C), suggesting that they are flatter and more adherent to the substratum. *Dictyostelium* cells have a traction force-dependent cytokinesis mode (cytokinesis B) [38]. 

**Figure 6:**
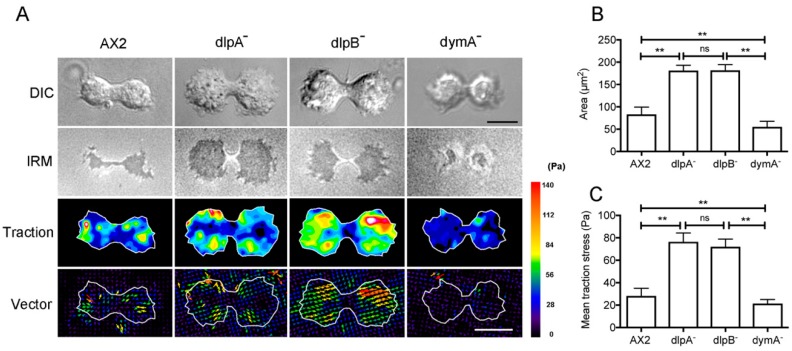
Dynamins contribute to cell-substratum adhesion and traction force. (**A**) Typical DIC images, interference reflection microscopy (IRM) images, traction maps, and traction vector maps of dividing cells (A×2, dlpA^-^, dlpB^-^, and dymA^-^). Each arrow in the traction vector map indicates both the magnitude and direction of the traction stress. The color code indicates the magnitude of the traction stresses; Bar, 10 µm. (**B**) Comparison of the cell-substratum adhesion area among these cells (*n* = 15, each). (**C**) Comparison of the mean traction stresses among these cells (*n* = 15, each). Data are presented as mean ± SD and analyzed by one-way ANOVA with Tukey’s multiple comparison test. ** *p* ≤ 0.001; ns, not significant, *p* > 0.05.

HS1 cells divide by traction forces exerted by the two daughter cells on the surface [18]. Dividing HS1 cells appear darker under phase-contrast microscopy and exert a larger traction force than the wild-type cells [22]. We speculated that the defects in the organization of contractile rings in dynamin mutant cells might affect the cell–substratum adhesion, traction force, and cytokinesis mode. Therefore, we simultaneously observed the cell–substratum adhesion and traction force using reflection interference (IR) and traction force (TF) microscopy. Figure 6A shows representative differential interferential contrast (DIC) images, IR images, traction maps, and vector maps.

The dark area in the IR micrograph was quantified as the cell–substratum adhesion area in each mutant cell (Figure 6B). The averaged adhesion areas of dlpA^-^ and dlpB^-^ cells were significantly larger than that of the wild-type cells, whereas the averaged adhesion area of dymA^-^ cells was significantly smaller than that of the wild-type cells.

Mean traction stresses of dlpA^-^ and dlpB^-^ cells were much higher than those of wild-type cells (Figure 6C). The vector map indicates that most of the traction stress was directed inward from both the polar regions in all dividing cells, which suggests that both the daughter halves migrate in the opposite directions, thereby exerting traction stress against the substratum towards the cell body. The mean traction stress of dymA^-^ cells was significantly lower than that of the wild-type cells.

Together, these results suggest that these dynamins contribute to the regulation of the cell-substratum adhesion and traction force.

### 3.8. DlpA Localizes at the Phagocytic Cup Independently of DlpB

**Figure 7:**
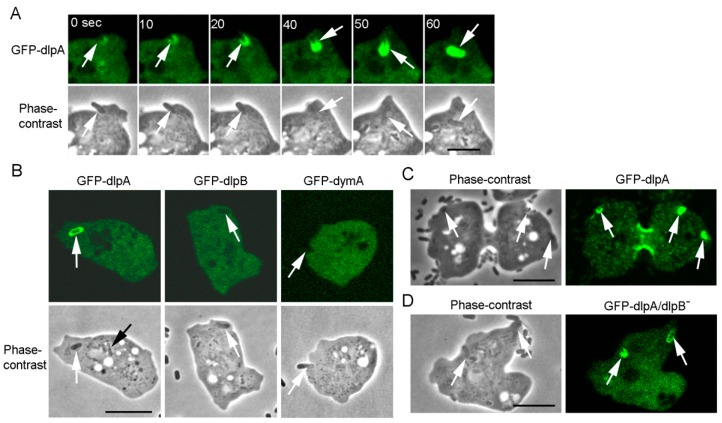
DlpA localizes at the phagocytic cups independently of dlpB. (**A**) A typical sequence of phagocytosis of a cell expressing GFP-dlpA. GFP-dlpA first appeared around the bacterium, and then encircled the endosome after it was engulfed (arrows). (**B**) Bacteria were added to each null mutant cell line expressing GFP-dlpA, GFP-dlpB, and GFP-dymA, independently. DlpB and dymA did not accumulate to the phagocytic cups in contrast to dlpA (white arrows). About 1 min after the bacteria were internalized, dlpA disappeared from the endosome (black arrow). (**C**) GFP-dlpA also localized at the phagocytic cups in dividing cells (arrows). Incidentally, GFP-dlpB did not localize at the phagocytic cups in dividing cells (data not shown). (**D**) Typical phase contrast and fluorescence images of a dlpB^-^ cell expressing GFP-dlpA. GFP-dlpA localized at the phagocytic cups independently of dlpB (arrows). Bars, 10 µm.

DlpA and dlpB may not always function cooperatively with each other. Interestingly, dlpA localized at phagocytic cups when cells internalized bacteria. Figure 7A shows typical sequential events during phagocytosis of a cell expressing GFP-dlpA. When the cell extended pseudopods around a bacterium and encircled and internalized it, GFP-dlpA localized around the bacterium. In contrast, neither dlpB nor dymA showed any localization at the phagocytic cups (Figure 7B, arrows). The dlpA was also observed at the phagocytic cups in the dividing cells (Figure 7C), suggesting that dlpA accumulates there independently of the cell cycle. Interestingly, GFP-dlpA localized at the phagocytic cups in dlpB^-^ cells (Figure 7D). Therefore, dlpA accumulation at the phagocytic cups is independent of dlpB.

## 4. Discussion

The present study provides the first report about the role of dlpB in *Dictyostelium* cells. We found that dlpB, along with dlpA and dymA, contributes to cytokinesis. The mutants deficient in these dynamins exhibited defects of cytokinesis in both suspension and adherent culture conditions. The cytokinesis defects were much more severe in suspension culture conditions, which is reminiscent of defects of myosin II null cells [39]. Myosin II also regulates the dynamics of the contractile ring, and the deletion of myosin II results in the failure of cytokinesis [40]. Dysfunction of the dynamic organization of the actin structure in the contractile ring of the dynamin mutant cells may also result in cytokinesis defects. The cytokinesis defects were found to be of similar extent in dlpA^-^, dlpB^-^, and dlpA^-^/dlpB^-^ double knockout cells. In addition, dlpA and dlpB were found to colocalize at the cleavage furrow from the early phase of furrowing till the final separation. TIRF microscopy revealed dlpB as small dots, where dlpA colocalized. Furthermore, dlpA and dlpB were co-precipitated in the co-immunoprecipitation experiments; dlpA did not localize at the cleavage furrow in dlpB^-^ cells and vice versa. DlpA and dlpB colocalized with actin filaments in the contractile ring, and latrunculin A treatment lost their localization. All these results strongly suggest that the hetero-oligomerization is required for these molecules to accumulate at the cleavage furrow. The hetero-oligomerization of dynamins has been recently identified in mitochondrion fission in *Entamoeba histolytica* [41].

The role of dymA in cytokinesis seems to be different from that of the dlpA and dlpB complex. Dividing dymA^-^ cells frequently formed a long intercellular bridge, and took much longer time for the final separation. Taken together with its localization at the intercellular bridge, dymA might contribute to the final separation.

Endocytosis has been implicated in cytokinesis. Continuous endocytosis is crucial for cytokinesis of zebrafish blastomeres; endocytosis inhibitors are known to block the separation of the daughter cells [5]. Clathrin null *Dictyostelium* cells have defects in cytokinesis [42]. *Dictyostelium* lvsA, which is involved in membrane trafficking, is required for cytokinesis [43]. Mutations in clathrin or dynamin also lead to cytokinesis failure in other organisms, such as *C. elegans*, *Drosophila*, and mammalian and plant cells [6,7,10,11,44,45]. These observations suggest that dynamin and clathrin may cooperatively participate in the endocytosis or membrane trafficking pathways required for cytokinesis. However, as dlpA and dlpB did not colocalize with clathrin, it is unlikely that they are directly involved in the clathrin-mediated endocytosis; they might be involved in other types of endocytosis. In zebrafish blastomeres, caveolae-mediated endocytosis, as well as clathrin-mediated endocytosis, contribute to cytokinesis [5]. However, we could not find any gene homologous to caveolin in the *Dictyostelium* genome.

Another possible role of the dlpA and dlpB complex in cytokinesis is the regulation of actin filaments in the contractile ring. The present results suggest that the hetero-oligomers of dlpA and dlpB directly or indirectly bind to the actin filaments and stabilize them. Dynamin I has been reported to directly bind to the actin filaments and remove the actin-capping protein, gelsolin, from barbed ends in vitro, thus allowing the elongation of the actin filaments [46]. Conversely, direct binding of short actin filaments to dynamin 1 enhances the oligomerization of dynamin 1 in vitro as well as in vivo [47]. A cross talk between actin and dynamin has also been reported in phagosome formation and closure [48]. The actin-binding site of dynamin 1 is located at amino acids 399–444 of the middle domain [46]. DymA and dymB have approximately 45% and 28% homology to this sequence in their middle domains, respectively; however, dlpA and dlpB have no middle domain equivalent to that of dynamin 1. The direct interaction between purified actin and *Dictyostelium* dynamins in vitro remains to be examined in the future.

Dynamins can also indirectly interact with actin filaments by binding to several actin-binding proteins, which is mediated by the proline, arginine-rich domain (PRD) at dynamin’s C-terminus [49]; nonetheless, the PRD domain is absent in *Dictyostelium* dynamins. Mammalian dynamin 1 binds to profilin I and II [50], which are essential for actin assembly. The complex of dynamin 1 and cortactin has been reported to stabilize actin filaments in the growth cones of the neuronal cells [51]. Dynamin 2 influences actin nucleation by the Arp2/3 complex and cortactin in vitro in a biphasic manner; low concentration of dynamin 2 enhances actin nucleation by Arp2/3 complex and cortactin, whereas a high concentration is inhibitory [52]. Dynamin 2 also modulates localization of Rac, a small GTPase that regulates actin cytoskeletons [53].

The fact that dlpA^-^ and dlpB^-^ cells showed a faster turnover of actin filaments than the wild-type cells indicates that these dynamins are likely to be responsible for antagonizing the filament severing activities, thereby helping to stabilize actin filaments in the contractile rings. Alternatively, dynamin may promote actin polymerization as described above [46] and maintain a constant length of actin filaments in the contractile rings. Myosin II is a candidate protein responsible for severing actin filaments in the contractile ring [31]. Cofilin and formin, in addition to myosin II, are known as the proteins responsible for regulating the length of actin filaments in the contractile rings in other organisms [54,55,56]. The cooperation between *Dictyostelium* dynamins and these proteins needs to be examined.

Another role of dlpA and dlpB is their involvement in the cell–substratum adhesion and traction force. It was previously reported that dymB^-^ cells are flatter on the substratum than the wild-type cells [14]. In the present study, we found that dlpA^-^ and dlpB^-^ cells have a wider adhesion area and higher traction force than the wild-type cells. However, dymA^-^ cells showed the opposite features. These dynamins may be involved in the formation of the focal adhesions, which anchor the actin filaments [57]. Although these *Dictyostelium* dynamins do not localize at the focal adhesions, dynamins have been reported to localize at the focal adhesions in mammalian cells [58,59]. Aberrant cell–substratum adhesion may result in cytokinesis defects of *Dictyostelium* dynamin null cells on a surface. How dynamins contribute to the cell adhesion and traction force remains to be clarified in the future.

Although dlpA and dlpB were mutually dependent on each other for their accumulation at the cleavage furrow (Figure 4B), dlpA accumulated at the phagocytic cup independently of dlpB. DlpA may function independently of dlpB in phagocytosis. DymA has been reported to localize at the phagosomes during their maturation but, unlike dlpA, dymA did not localize at the phagocytic cup at an early stage of phagocytosis [60]. The regulatory mechanisms underlying the localization of dlpA and dymA at the cleavage furrow or the phagocytic apparatus remain elusive. Such questions also need further attention in the future.

**Figure 8:**
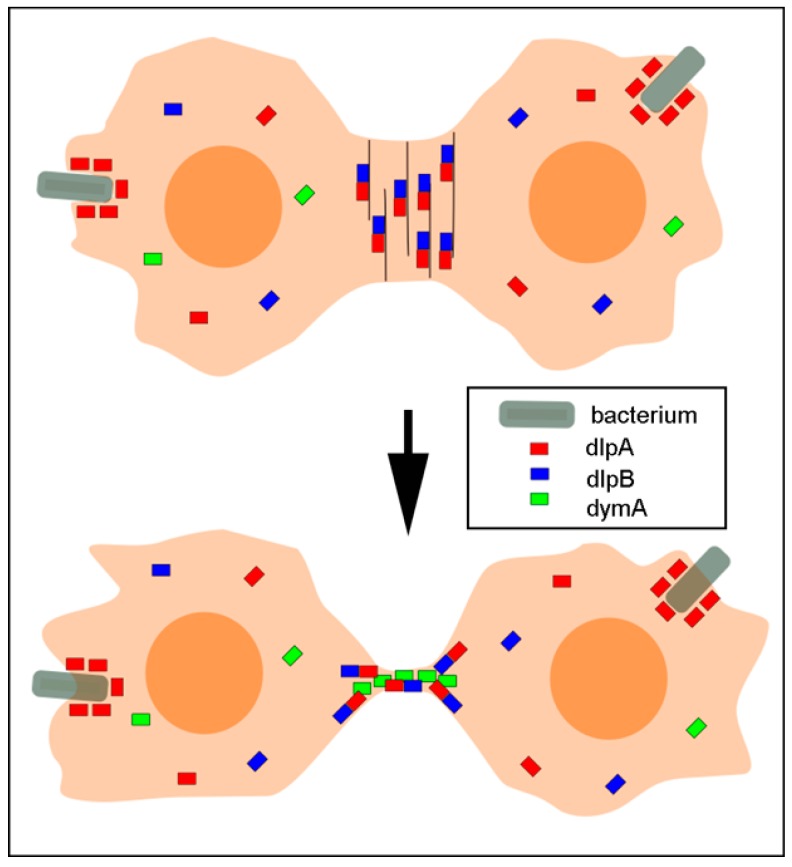
A summary of the localization of three dynamins. Dynamics of dlpA (red small bars), dlpB (blue small bars), and dymA (green small bars) during cytokinesis are shown. DlpA and dlpB form hetero-oligomers and associate with the actin filaments in the contractile ring. DlpA also localizes at the phagocytic cup surrounding a bacterium (gray bars) independently of dlpB. DymA localizes at the intercellular bridge between the daughter cells.

Figure 8 summarizes the localization of the three dynamins. DlpA and dlpB form hetero-oligomers and associate with and stabilize the actin filaments in the contractile ring. DymA localizes at the intercellular bridge in the last stage of cytokinesis. Therefore, we conclude that dlpB contributes to cytokinesis cooperatively with dlpA and dymA.

## References

[1] Heymann J.A., Hinshaw J.E. (2009). Dynamins at a glance. J. Cell Sci..

[2] Jimah J.R., Hinshaw J.E. (2019). Structural Insights into the Mechanism of Dynamin Superfamily Proteins. Trends Cell Biol..

[3] Chircop M., Sarcevic B., Larsen M.R., Malladi C.S., Chau N., Zavortink M., Smith C.M., Quan A., Anggono V., Hains P.G. (2011). Phosphorylation of dynamin II at serine-764 is associated with cytokinesis. Biochim. Biophys. Acta..

[4] Ishida N., Nakamura Y., Tanabe K., Li S.A., Takei K. (2011). Dynamin 2 associates with microtubules at mitosis and regulates cell cycle progression. Cell Struct. Funct..

[5] Feng B., Schwarz H., Jesuthasan S. (2002). Furrow-specific endocytosis during cytokinesis of zebrafish blastomeres. Exp. Cell Res..

[6] Thompson H.M., Skop A.R., Euteneuer U., Meyer B.J., McNiven M.A. (2002). The large GTPase dynamin associates with the spindle midzone and is required for cytokinesis. Curr. Biol..

[7] Pelissier A., Chauvin J.P., Lecuit T. (2003). Trafficking through Rab11 endosomes is required for cellularization during Drosophila embryogenesis. Curr. Biol..

[8] Gu X., Verma D.P. (1996). Phragmoplastin, a dynamin-like protein associated with cell plate formation in plants. Embo J..

[9] Ahn G., Kim H., Kim D.H., Hanh H., Yoon Y., Singaram I., Wijesinghe K.J., Johnson K.A., Zhuang X., Liang Z. (2017). SH3 Domain-Containing Protein 2 Plays a Crucial Role at the Step of Membrane Tubulation during Cell Plate Formation. Plant Cell.

[10] Kang B.H., Busse J.S., Bednarek S.Y. (2003). Members of the Arabidopsis dynamin-like gene family, ADL1, are essential for plant cytokinesis and polarized cell growth. Plant Cell.

[11] Miyagishima S.Y., Kuwayama H., Urushihara H., Nakanishi H. (2008). Evolutionary linkage between eukaryotic cytokinesis and chloroplast division by dynamin proteins. Proc. Natl. Acad. Sci. USA.

[12] Arakaki Y., Fujiwara T., Kawai-Toyooka H., Kawafune K., Featherston J., Durand P.M., Miyagishima S.Y., Nozaki H. (2017). Evolution of cytokinesis-related protein localization during the emergence of multicellularity in volvocine green algae. BMC Evol. Biol..

[13] Schlimpert S., Wasserstrom S., Chandra G., Bibb M.J., Findlay K.C., Flärdh K., Buttner M.J. (2017). Two dynamin-like proteins stabilize FtsZ rings during Streptomyces sporulation. Proc. Natl. Acad. Sci. USA.

[14] Rai A., Nothe H., Tzvetkov N., Korenbaum E., Manstein D.J. (2011). Dictyostelium dynamin B modulates cytoskeletal structures and membranous organelles. Cell Mol. Life Sci..

[15] Schimmel B.G., Berbusse G.W., Naylor K. (2012). Mitochondrial fission and fusion in Dictyostelium discoideum: A search for proteins involved in membrane dynamics. BMC Res. Notes.

[16] Wienke D.C., Knetsch M.L., Neuhaus E.M., Reedy M.C., Manstein D.J. (1999). Disruption of a dynamin homologue affects endocytosis, organelle morphology, and cytokinesis in Dictyostelium discoideum. Mol. Biol. Cell.

[17] Masud A.Y.K.M., Tsujioka M., Miyagishima S., Ueda M., Yumura S. (2013). Dynamin contributes to cytokinesis by stabilizing actin filaments in the contractile ring. Genes Cells.

[18] Neujahr R., Heizer C., Gerisch G. (1997). Myosin II-independent processes in mitotic cells of Dictyostelium discoideum: Redistribution of the nuclei, re-arrangement of the actin system and formation of the cleavage furrow. J. Cell Sci..

[19] Taira R., Yumura S. (2017). A novel mode of cytokinesis without cell-substratum adhesion. Sci. Rep..

[20] Tanaka Y., Jahan M.G.S., Kondo T., Nakano M., Yumura S. (2019). Cytokinesis D is Mediated by Cortical Flow of Dividing Cells Instead of Chemotaxis. Cells.

[21] Uyeda T.Q., Nagasaki A. (2004). Variations on a theme: The many modes of cytokinesis. Curr. Opin. Cell Biol..

[22] Jahan M.G.S., Yumura S. (2017). Traction force and its regulation during cytokinesis in Dictyostelium cells. Eur. J. Cell Biol..

[23] Pollard T.D. (2017). Nine unanswered questions about cytokinesis. J. Cell Biol.

[24] Yumura S., Matsuzaki R., Kitanishi-Yumura T. (1995). Introduction of macromolecules into living Dictyostelium cells by electroporation. Cell Struct. Funct..

[25] Yumura S. (2016). A novel low-power laser-mediated transfer of foreign molecules into cells. Sci. Rep..

[26] Linkner J., Nordholz B., Junemann A., Winterhoff M., Faix J. (2012). Highly effective removal of floxed Blasticidin S resistance cassettes from Dictyostelium discoideum mutants by extrachromosomal expression of Cre. Eur. J. Cell Biol..

[27] Manstein D.J., Titus M.A., De Lozanne A., Spudich J.A. (1989). Gene replacement in Dictyostelium: Generation of myosin null mutants. Embo J..

[28] Okazaki K., Yumura S. (1995). Differential association of three actin-bundling proteins with microfilaments in Dictyostelium amoebae. Eur. J. Cell Biol..

[29] Yumura S., Yoshida M., Betapudi V., Licate L.S., Iwadate Y., Nagasaki A., Uyeda T.Q., Egelhoff T.T. (2005). Multiple myosin II heavy chain kinases: Roles in filament assembly control and proper cytokinesis in Dictyostelium. Mol. Biol. Cell.

[30] Yumura S., Mori H., Fukui Y. (1984). Localization of actin and myosin for the study of ameboid movement in Dictyostelium using improved immunofluorescence. J. Cell Biol..

[31] Yumura S., Ueda M., Sako Y., Kitanishi-Yumura T., Yanagida T. (2008). Multiple mechanisms for accumulation of myosin II filaments at the equator during cytokinesis. Traffic.

[32] Yumura S. (2001). Myosin II dynamics and cortical flow during contractile ring formation in Dictyostelium cells. J. Cell Biol..

[33] Kong L., Sochacki K.A., Wang H., Fang S., Canagarajah B., Kehr A.D., Rice W.J., Strub M.-P., Taraska J.W., Hinshaw J.E. (2018). Cryo-EM of the dynamin polymer assembled on lipid membrane. Nature.

[34] Podolski J.L., Steck T.L. (1988). Association of deoxyribonuclease I with the pointed ends of actin filaments in human red blood cell membrane skeletons. J. Biol. Chem..

[35] Kabsch W., Mannherz H.G., Suck D., Pai E.F., Holmes K.C. (1990). Atomic structure of the actin:DNase I complex. Nature.

[36] Yumura S., Itoh G., Kikuta Y., Kikuchi T., Kitanishi-Yumura T., Tsujioka M. (2013). Cell-scale dynamic recycling and cortical flow of the actin-myosin cytoskeleton for rapid cell migration. Biol. Open.

[37] Courtemanche N., Pollard T.D., Chen Q. (2016). Avoiding artefacts when counting polymerized actin in live cells with LifeAct fused to fluorescent proteins. Nat. Cell Biol..

[38] Uyeda T.Q., Kitayama C., Yumura S. (2000). Myosin II-independent cytokinesis in Dictyostelium: Its mechanism and implications. Cell Struct. Funct..

[39] Zang J.H., Cavet G., Sabry J.H., Wagner P., Moores S.L., Spudich J.A. (1997). On the role of myosin-II in cytokinesis: Division of Dictyostelium cells under adhesive and nonadhesive conditions. Mol. Biol. Cell.

[40] Yumura S., Uyeda T.Q. (2003). Myosins and cell dynamics in cellular slime molds. Int. Rev. Cytol..

[41] Makiuchi T., Santos H.J., Tachibana H., Nozaki T. (2017). Hetero-oligomer of dynamin-related proteins participates in the fission of highly divergent mitochondria from Entamoeba histolytica. Sci. Rep..

[42] Gerald N.J., Damer C.K., O’Halloran T.J., De Lozanne A. (2001). Cytokinesis failure in clathrin-minus cells is caused by cleavage furrow instability. Cell Motil. Cytoskelet..

[43] Kwak E., Gerald N., Larochelle D.A., Vithalani K.K., Niswonger M.L., Maready M., De Lozanne A. (1999). LvsA, a protein related to the mouse beige protein, is required for cytokinesis in Dictyostelium. Mol. Biol. Cell.

[44] Konopka C.A., Schleede J.B., Skop A.R., Bednarek S.Y. (2006). Dynamin and cytokinesis. Traffic.

[45] Smith C.M., Chircop M. (2012). Clathrin-Mediated Endocytic Proteins are Involved in Regulating Mitotic Progression and Completion. Traffic.

[46] Gu C., Yaddanapudi S., Weins A., Osborn T., Reiser J., Pollak M., Hartwig J., Sever S. (2010). Direct dynamin-actin interactions regulate the actin cytoskeleton. Embo J..

[47] Gu C., Chang J., Shchedrina V.A., Pham V.A., Hartwig J.H., Suphamungmee W., Lehman W., Hyman B.T., Bacskai B.J., Sever S. (2014). Regulation of dynamin oligomerization in cells: The role of dynamin-actin interactions and its GTPase activity. Traffic.

[48] Marie-Anais F., Mazzolini J., Herit F., Niedergang F. (2016). Dynamin-Actin Cross Talk Contributes to Phagosome Formation and Closure. Traffic.

[49] Sever S., Chang J., Gu C. (2013). Dynamin rings: Not just for fission. Traffic.

[50] Witke W., Podtelejnikov A.V., Di Nardo A., Sutherland J.D., Gurniak C.B., Dotti C., Mann M. (1998). In mouse brain profilin I and profilin II associate with regulators of the endocytic pathway and actin assembly. Embo J..

[51] Yamada H., Abe T., Satoh A., Okazaki N., Tago S., Kobayashi K., Yoshida Y., Oda Y., Watanabe M., Tomizawa K. (2013). Stabilization of actin bundles by a dynamin 1/cortactin ring complex is necessary for growth cone filopodia. J. Neurosci..

[52] Schafer D.A., Weed S.A., Binns D., Karginov A.V., Parsons J.T., Cooper J.A. (2002). Dynamin2 and cortactin regulate actin assembly and filament organization. Curr. Biol..

[53] Schlunck G., Damke H., Kiosses W.B., Rusk N., Symons M.H., Waterman-Storer C.M., Schmid S.L., Schwartz M.A. (2004). Modulation of Rac localization and function by dynamin. Mol. Biol Cell.

[54] Chen Q., Pollard T.D. (2011). Actin filament severing by cofilin is more important for assembly than constriction of the cytokinetic contractile ring. J. Cell Biol..

[55] Schramm A.C., Hocky G.M., Voth G.A., Blanchoin L., Martiel J.L., De La Cruz E.M. (2017). Actin Filament Strain Promotes Severing and Cofilin Dissociation. Biophys. J..

[56] Watanabe S., Ando Y., Yasuda S., Hosoya H., Watanabe N., Ishizaki T., Narumiya S. (2008). mDia2 induces the actin scaffold for the contractile ring and stabilizes its position during cytokinesis in NIH 3T3 cells. Mol. Biol. Cell.

[57] Uchida K.S., Yumura S. (2004). Dynamics of novel feet of Dictyostelium cells during migration. J. Cell Sci..

[58] Kruchten A.E., McNiven M.A. (2006). Dynamin as a mover and pincher during cell migration and invasion. J. Cell Sci..

[59] Ochoa G.C., Slepnev V.I., Neff L., Ringstad N., Takei K., Daniell L., Kim W., Cao H., McNiven M., Baron R. (2000). A functional link between dynamin and the actin cytoskeleton at podosomes. J. Cell Biol..

[60] Gueho A., Bosmani C., Gopaldass N., Molle V., Soldati T., Letourneur F. (2016). Dictyostelium EHD associates with Dynamin and participates in phagosome maturation. J. Cell Sci..

